# Lightness in a Flash: Effect of Exposure Time on Lightness Perception

**DOI:** 10.1177/2041669520983830

**Published:** 2020-12-25

**Authors:** Sae Kaneko, Alan Gilchrist

**Affiliations:** Frontier Research Institute for Interdisciplinary Sciences, Tohoku University, Miyagi, Japan; Research Institute of Electrical Communication, Tohoku University, Miyagi, Japan; Psychology Department, Rutgers University, Newark, New Jersey, United States

**Keywords:** exposure time, illusion, lightness/brightness, spatial context

## Abstract

A gray target can appear lighter or darker depending on its surrounding spatial context. We examined the effect of exposure time on three such examples (simultaneous lightness contrast, dungeon illusion, and the two-room arrangement), finding very different results with exposure time as brief as 15 ms: the simultaneous lightness contrast was much stronger, the effect of the dungeon illusion was reversed, and the lightness difference between the two isoluminant patches in the two-room arrangement disappeared. These suggest that local luminance ratios dominate lightness perception in a brief flash.

That we can perceive a white piece of paper in the shade as white or a black object in the bright illumination as black is one of our most remarkable visual abilities. Of course, this is not always true; in some rare situations, we can see a gray object as white or as black. One of the most simple yet fascinating examples is simultaneous lightness contrast (Figure 1A). In the classic textbook version, two identical gray squares set in side by side white and black backgrounds respectively look quite different in lightness; the one on black looks lighter than the other.

**Figure 1.**
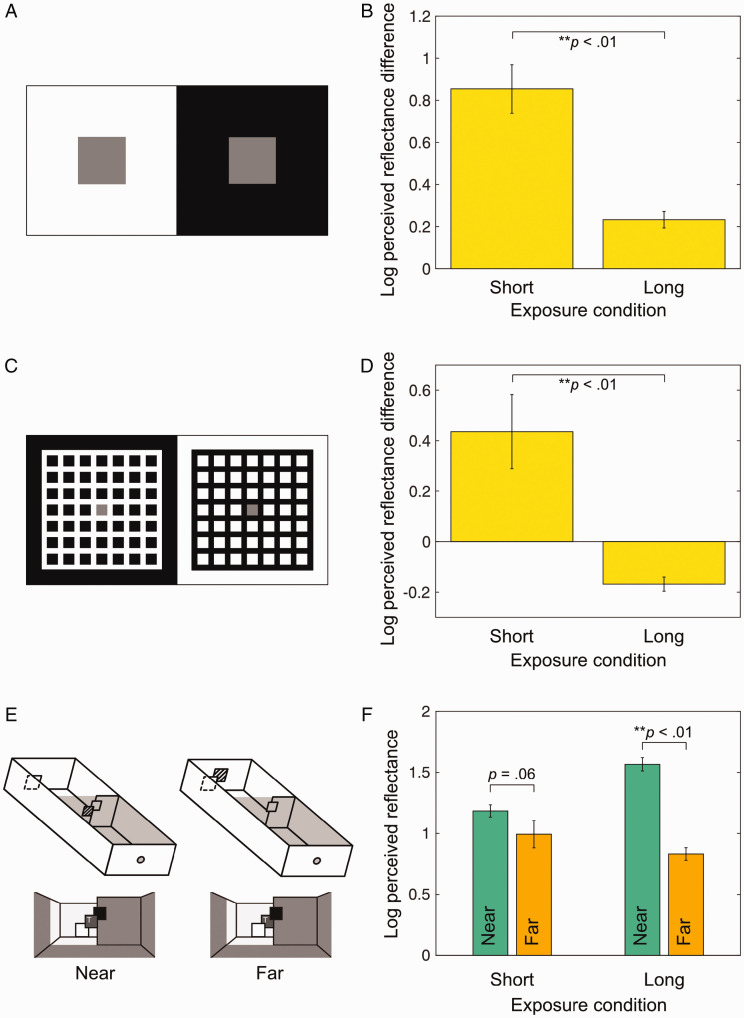
Stimuli and corresponding results (mean ± 1 SEM). (A) Simultaneous lightness contrast and (B) difference in log perceived reflectance between the two squares (right minus left). (C) Bressan’s dungeon illusion and (D) difference in log perceived reflectance between the two squares (right minus left). E: Schematic illustration of two-room arrangement with the lid covering the near room omitted from illustration. The target (shown here as cross hatched) was located either on the near wall (left) or on the far wall (right). F: Log perceived reflectance in four conditions.

Early theories of lightness perception often relied on local contrast as the calculation basis of lightness. For instance, Wallach (1948) claimed that lightness perception was determined by the luminance ratio between a target and its immediate background.

Although it may predict the simultaneous contrast illusion, his theory fails to explain many other phenomena. For example, in Bressan’s (2001) dungeon illusion (Figure 1C), two identical gray patches are locally surrounded by white and black but the one surrounded by black now looks darker, not lighter; in other words, the illusory effect is opposite to the simultaneous lightness contrast. Obviously, Wallach’s luminance ratio theory cannot account for this. Another example is Gilchrist’s (1977) two-room arrangement. Gilchrist demonstrated with this setup that a given target could look white or black depending on its perceived location; when it appeared to be in the nearer dark room it appeared white and when it appeared to be in the far lighted room it appeared black. Because the retinal image was essentially the same in both conditions, the targets should appear equal in lightness, according to Wallach. Yet the fact that the targets appeared very different clearly indicates that lightness perception takes the three-dimensional layout into account. Gilchrist later established his anchoring theory of lightness (Gilchrist et al., 1999), which provides an account of all the three aforementioned phenomena.

Now, none of these theories of lightness perception involves the temporal dimension. They imply that lightness perception does not vary with the stimulus duration. However, Kaneko and Murakami (2012) demonstrated that the simultaneous contrast illusion is much stronger when the stimulus duration is 10 ms compared to 500 ms. They also showed that a spatial gap between the target and the background greatly reduced the illusion when the duration was 10 ms, but not when the duration was 500 ms. These results seem to indicate that lightness perception does vary with duration and the role of adjacent luminance ratio is much larger in a brief flash. Is this only true for a highly simplified figures on a computer monitor (as in Kaneko & Murakami, 2012) or does the same apply for more complex, realistic stimuli?

We took three stimuli, namely, simultaneous lightness contrast, Bressan’s dungeon illusion, and the two-room arrangement (all made with paper; see Figure 1A, C, and E, respectively) to explore the effect of duration on lightness perception. We chose these displays as our sample for the following reasons. For many years, lightness was attributed to lateral inhibitory processes operating on the retinal image. This view was later overthrown by empirical results showing that lightness depends on factors like perceived depth and perceptual grouping. The following three, while not exhaustive, are considered fundamental to any lightness theory. Simultaneous lightness contrast is a basic lightness effect. The dungeon illusion is probably the strongest of the reverse contrast phenomena in which the effect of a background completely surrounding a target is opposite to that of simultaneous contrast. The Gilchrist two-room experiment produced some of the strongest effects of depth on lightness.

We achieved a very brief viewing time of 15 ms with an old-fashioned Prontor camera shutter. All three stimuli were set up in a viewing box with a small (monocular) viewing window at one end. Observers selected the most similar lightness from a 16-chip (2.0 to 9.5) Munsell chart placed in room illumination outside the viewing box. There was no limit to viewing time in the long condition, or number of observer-triggered exposures in the short condition.

Thirty observers matched both targets in the simultaneous contrast display as did 30 others in the dungeon illusion (Figure 1A and C), with half of each group given short exposure and half long. Figures 1B and D show the results in log perceived reflectance. As is clear in Figure 1B, simultaneous lightness contrast was present for both conditions, but almost 4 times stronger in short exposure compared with long exposure, which confirmed Kaneko and Murakami (2012). While we successfully replicated Bressan’s reverse-contrast effect for long exposure, we found a surprising reversal of the illusion for short exposure. In a flash, the target surrounded by the white grid now looked darker not lighter.

In the two-room arrangement (Figure 1E), unlike the original study (Gilchrist, 1977), the target was physically on the near wall or the far wall, but the principle stayed the same. Target luminance and retinally neighboring patches and backgrounds were identical in both conditions. Sixty observers served here, 30 observers in the near condition and 30 others in the far condition, with half of each group given each exposure. In the long condition, the near target appeared light gray and the far target as black, confirming Gilchrist (1977). However, this difference disappeared or was greatly reduced in the short condition (Figure 1F). It was not that they could not see the stimuli in depth; all but one observers we asked correctly located the target in depth (near or far). It appears that the depth simply did not affect lightness perception for this condition.

All three experiments point to a clear difference in lightness perception between the normal (unlimited) and the extremely limited viewing conditions. Under long exposure, perceived three-dimensional arrangement and perceptual grouping were critical, as suggested by previous studies (Bressan, 2001; Gilchrist, 1977). In a brief flash, perception was apparently governed by adjacent luminance ratios in the retinal image, as per Wallach’s theory. Effects of depth or grouping seemed to require more processing time.

Wallach’s luminance ratio rule may not generalize to other lightness effects seen in a flash. We hope further work should clarify the underlying process of lightness perception which may develop over time.
